# Philosophy of Medical Investigation*Oration on the Guidance of a Sound Philosophical Spirit in the Investigation of Medical Science, read before the Cincinnati Medical Society, January 4, 1837. By John P. Harrison, M. D., Professor of Materia Medica in the Medical Department of the Cincinnati College. Printed by order of the Society.

**Published:** 1837

**Authors:** 


ART. X.—THE PHILOSOPHY OF MEDICAL INVESTIGATION.*

THE Cincinnati Medical Society was chartered in the year 1831. Throughout the winter its meetings are weekly. Pa-

* Oration on the Guidance of a Sound Philosophical Spirit in the Investigation of Medical Science, read before the Cincinnati Medical Society, January 4, 1837. By John P. Harrison, M. D., Professor of Materia Medica in the Medical Department of the Cincinnati College. Printed by order of the Society.

pers are read and discussed, and its debates are occasionally animated, comprehensive, and instructive. In the autumn of 1836, Professor Harrison was elected to deliver its anniversary oration, the title of which we have just transcribed. The Professor is *now* one of the editors and proprietors of this journal, but as a notice of his discourse was crowded out of the last number, when he had no connexion with the journal, a colleague may be excused for saying of it still, what he would then have said, that it abounds in just views and most salutary advice. We shall take, at random, a page which denounces, in appropriate terms, the practice of men of genius, in manufacturing premature generalizations.

"Owing to excessive facility of belief, the crude notions of Brown, became at one time the pathological ground upon which diseases were treated. This bold, original, speculative genius, perémptorily announced, that under his plastic hand, medicine, which had hitherto been mainly conjectural, was now to assume the grandeur and solidity of a certain, demonstrable, enduring science. And how was this most desirable result to be obtained? By the creations of his own imaginative mind, independent of observation and experience. Thus did he exemplify the justness of the critical animadversion of Bacon, directed against the vicious mode of philosophizing which obtained among certain ancient sophists, whom he designated as 'men who searched for truth in their own little minds, not in the great world without them.' In more recent times, the ultra views of Broussais have been too eagerly received, indiscriminately adopted, and acted upon, by many medical men. It augurs well for our science that the confidence of the profession in such all-embracing views of disease is rapidly subsiding. Even the students of medicine, who attend our medical schools, from the prevalent tone of thinking in society, and from the lessons of a correct mental discipline, are every day passing adjudication, in their own minds, in favor of those teachers who, with their doctrinal expositions of disease, interweave practical illustrations. We cultivate a directly practical art—we pursue, as physicians, a calling which brings us every day in direct contact with the lives of our fellow-men. Any scheme of doctrines which stands alien and apart, in solitary grandeur, like the pyramids in the desert, from these practical applications to which every truth in medical science ought to be made to bend, should be looked at with philosophical distrust by every mind, intent on the discovery of truth, and that desires not to lavish its powers upon barren speculation and sterile generalities. I am aware that students of medicine are not always capable of appreciating the discussion of general principles, although such general principles are absolutely essential to a just and philosophical understanding of disease. Let general principles be thoroughly discussed, till the light of a most lucid exposition be made to pour its lustre upon those doctrines, which lie at the very foundation of all enlightened conceptions of morbid action. But should there arise in any part of the wide field of medical doctrines, a destitution of those proofs upon which the teacher should rely for the verity of his doctrinal statements;—should he, for instance, lay down a certain pathology of disease as true, and then, after such an abstract position fail to adduce proofs from the symptoms, treatment, and post mortem appearances of persons dying with such disease, that his theoretic principles were just—then let him forego the delights of such entertainment of fancy—and either quickly supply himself with better views, or candidly confess his ignorance. 'It ought to be inculcated upon all men,' says a French writer, 'that next to the positive knowledge of things which may be known, the most important science is to know how to be ignorant. 'I don't know,' ought to be the frequent answer of all teachers to their pupils, to accustom them to make the same answer without feeling ashamed.

True philosophical scepticism is never ashamed to confess the want of knowledge, where there is no proof offered. It can wait—it will wait—most patiently, for that proof, and not till it is obtained, does it abandon the sure ground of doubt, of inquiry, of diligent search, to move forward to the higher ground of confiding trust. And this leads me to expatiate a little on another trait of that philosophical spirit which should guide all our medical investigations—it is the love of truth."

We hope the Society will continue its labors with an understanding zeal, and that the first-born will be followed by a numerous progeny, equally worthy of its name and elevated objects. D.

